# Animal behaviour on the move: the use of auxiliary information and semi-supervision to improve behavioural inferences from Hidden Markov Models applied to GPS tracking datasets

**DOI:** 10.1186/s40462-023-00401-5

**Published:** 2023-07-24

**Authors:** Sarah Saldanha, Sam L. Cox, Teresa Militão, Jacob González-Solís

**Affiliations:** 1 Institut de Recerca de la Biodiversitat (IRBio), Universitat de Barcelona (UB), Barcelona, Spain; 2 Dept Biologia Evolutiva, Ecologia i Ciències Ambientals, Universitat de Barcelona, Av Diagonal 643, Barcelona, 08028 Spain; 3 Centre National d’Études Spatiales (CNES), Toulouse, 31400 France; 4 MARBEC, Univ Montpellier, CNRS, Ifremer, IRD, Sète, France; 5 Institut de Recherche pour le Développement (IRD), Sète, France; 6 MaREI Centre, University College Cork, Cork, Ireland

**Keywords:** Semi-supervision, HMM, Behavioural classification, Opportunistic foraging, Tropical oceans, Behavioural modes, Animal movement

## Abstract

**Background:**

State-space models, such as Hidden Markov Models (HMMs), are increasingly used to classify animal tracks into behavioural states. Typically, step length and turning angles of successive locations are used to infer where and when an animal is resting, foraging, or travelling. However, the accuracy of behavioural classifications is seldom validated, which may badly contaminate posterior analyses. In general, models appear to efficiently infer behaviour in species with discrete foraging and travelling areas, but classification is challenging for species foraging opportunistically across homogenous environments, such as tropical seas. Here, we use a subset of GPS loggers deployed simultaneously with wet-dry data from geolocators, activity measurements from accelerometers, and dive events from Time Depth Recorders (TDR), to improve the classification of HMMs of a large GPS tracking dataset (478 deployments) of red-billed tropicbirds (*Phaethon aethereus*), a poorly studied pantropical seabird.

**Methods:**

We classified a subset of fixes as either resting, foraging or travelling based on the three auxiliary sensors and evaluated the increase in overall accuracy, sensitivity (true positive rate), specificity (true negative rate) and precision (positive predictive value) of the models in relation to the increasing inclusion of fixes with known behaviours.

**Results:**

We demonstrate that even with a small informed sub-dataset (representing only 9% of the full dataset), we can significantly improve the overall behavioural classification of these models, increasing model accuracy from 0.77 ± 0.01 to 0.85 ± 0.01 (mean ± sd). Despite overall improvements, the sensitivity and precision of foraging behaviour remained low (reaching 0.37 ± 0.06, and 0.06 ± 0.01, respectively).

**Conclusions:**

This study demonstrates that the use of a small subset of auxiliary data with known behaviours can both validate and notably improve behavioural classifications of state space models of opportunistic foragers. However, the improvement is state-dependant and caution should be taken when interpreting inferences of foraging behaviour from GPS data in species foraging on the go across homogenous environments.

**Supplementary Information:**

The online version contains supplementary material available at 10.1186/s40462-023-00401-5.

## Background

Inferring behaviour from animal movements is crucial to understand relationships between species and their environments [1, 2] or potential human-wildlife conflicts [3–5]. Over the last three decades, advances in biologging technology through the creation of smaller, cheaper and more sophisticated and accurate sensors, have facilitated rapid developments in the field of movement ecology, allowing for the study of movement in a wide array of species and environments (e.g. [6]). In tandem, several statistical methods and modelling approaches have been developed which mathematically analyse step length (the distance between consecutive positions), angle, tortuosity, and other traits of a trajectory to infer what segments of an animal’s track are spent in specific behaviours based on knowledge of their locomotion and ecology [7]. This can be particularly useful for conservation and management [8], enabling the identification and protection of areas important for animal ecology, such as those associated with foraging [9, 10], and/or resting [11, 12]. However, whilst the study of animal movement is progressing rapidly, transforming tracking data into meaningful behavioural states still remains a challenge for many species.

Typically, attempts to segment tracks into behaviour use the step length and tortuosity of animal movements, acquired by transforming data from GPS/Argos loggers into a bivariate series of step lengths and turning angles [13]. Based on these values, tracks are then segmented into two or three behavioural states: foraging and travelling, and if anticipated, resting. To differentiate foraging from travelling, inference often relies on the concepts of Area Restricted Search (ARS) and Optimal Foraging Theory (OFT). ARS predicts that when resources are patchily distributed, foraging is concentrated in high density areas, within which there is a decrease in step length and an increase in turning angle rate [14]. Outside of these foraging patches, OFT predicts that animals will minimise time in transit to, from, and between foraging areas by taking the most direct route over unsuitable environments, resulting in fast,directed movements [15]. The identification of rest is often associated with a long period without movement in terrestrial environments or with movement associated with drift in aquatic environments [11, 12]. However, while several methods are commonly used to infer behaviour from GPS tracks, their results are rarely cross-validated, and when they are, show a disparate ability to correctly predict behavioural states (S1).

While some differences in model performance among studies can be attributed to the type of model and/or validation method [16–20], performance is highly dependant on how distinct behaviour-specific movement patterns are [16, 18, 20–22]. For example, in heterogeneous systems, where resources are patchily distributed in space and time in a predictable manner, animals typically follow the concepts of ARS and OFT, using commuting trips to actively seek out rich foraging patches while quickly bypassing nutrient poor areas, resulting in a clear separation between the movement patterns of travelling and foraging [17, 23]. However, in homogeneous systems, where resources are more evenly and often unpredictably distributed in space and time, species may adopt a more opportunistic approach and undertake looping trips, where foraging is sporadic and short-lived, termed foraging on the go [24–26]. In this case, models may struggle to separate foraging movements from travelling, resulting in high levels of misclassification. Difficulties in inference may be further exasperated when both resting and foraging take place at short step length or when the turning angle of resting is artificially high because of GPS error [27–29]. Limitations have been noted across a variety of modelling methods including Hidden Markov Models (HMMs) [28], Expectation-maximization binary clustering (EmbC) [26, 28], Residence in Space and Time (RST) [30], and First Passage Time (FPT) [29]. As a result, post-hoc adjustments are applied to improve model performance, either by pooling locations classified as resting and intensive search together [28], re-classifying foraging locations with step lengths representing speeds below those of local currents (1 m/s) as resting [30] or eliminating locations with short step lengths altogether before running the analysis [29]. However, the predictions of these models, both pre- and post-adjustments, are usually evaluated visually, and without cross-validation with other datasets making it difficult to measure the benefits of these changes (S1).

Model performance can be improved by incorporating additional information on what an animal is doing from auxiliary sensors. For example, wet-dry sensors (WD) can distinguish when an animal is immersed in salt water [3, 23], Time Depth Recorders (TDR) can be used to detect dives below a specific threshold [31] and high frequency tri-axial accelerometers can provide unprecedented information on fine-scale movements resulting in inferences that go as far as separating individual prey-capture attempts [17, 32–34]. Data acquired from these sensors can be incorporated into behavioural models, allowing for more accurate classification. Although several modelling techniques can be used to incorporate these data, HMMs have drawn particular attention due to their relatively high accuracy [22, 34], their robustness at lower GPS resolution [16, 20, 22], and the development of the flexible user-friendly R packages that can incorporate information from additional data streams, even when collected at different time resolutions (e.g. ‘moveHMM’ and ‘momentuHMM’; [33, 35, 36]). Nonetheless, the use of auxiliary sensors is often limited by their cost, size, and weight, and so they often only comprise a small fraction of a full GPS tracking dataset, and cannot easily be incorporated as additional datastreams [33]. For this reason, many studies limit their use to validate behaviours identified from GPS positions, instead of directly using these data to improve the model classifications themselves (e.g. [23, 37]).

When a small auxiliary sensor dataset is present, one potential solution is manually setting associated positions to a given inferred behaviour, and then use these positions to semi-supervise the model behavioural classification of the rest of the data-set, with an aim to improve the models’ overall accuracy. In this study, we aim to assess whether the addition of information from auxiliary sensors can improve behavioural inference in animals mainly performing looping trips through relatively homogeneous environments, such as seabirds foraging in tropical waters. We use a large GPS tracking dataset of a tropical seabird species, the red-billed tropicbird (*Phaethon aethereus*), of which a subset was double tagged with a combination of accelerometers, wet-dry sensors, and/or TDR sensors. From these auxiliary sensors, we determine informed positions of resting, foraging, and travelling and use these to semi-supervise the fitting of an HMM predominantly based on movement metrics between GPS fixes. Specifically, by incorporating additional auxiliary sensors to GPS tracking, we assess whether (1) model accuracy in identifying behavioural states improves with an increasing percentage of supervision; (2) the improvement in the inference is homogeneous across the three basic behavioural states, i.e. resting, foraging and travelling, and (3) this improvement saturates or could theoretically achieve behavioural inference levels comparable to those obtained for species using commuting trips. It is hoped that outputs from this study can direct researchers in the deployment of specific tracking regimes to yield the most accurate identification of behaviour from animal movement and to will limit errors that can contaminate future analyses,such as the identification of areas of ecological importance for species.

## Methods

### Fieldwork

Fieldwork took place at 7 colonies dispersed across 2 islands (Boavista and Sal) and 2 islets (Cima and Raso) in Cabo Verde between 2017 and 2021. While fieldwork on Sal and Boavista islands was almost continuous during this time, work on the islets was restricted to campaigns of a few months each until 2020, after which work on Cima Islet was nearly continuous, and discontinued on Raso.

Red-billed tropicbirds were captured on their nests during incubation or early chick-rearing, and equipped with a combination of CatLog Gen2 GPS, Axytrek loggers (which records GPS, tri-axial accelerometer, and time-depth information), and/or Migrate Technology geolocators (GLS) with a wet-dry sensor (salt water immersion logger). The GPS loggers used weighed 18 g (2.9% of mean tropicbird weight (630 g ± 55, n = 1297 individuals) and were programmed to record GPS positions every 5 min. Axytrack loggers weighed 17 g (2.6% of tropicbird weight) and recorded GPS, acceleration and pressure data at 5-minute, 25 Hz and 1s intervals, respectively. The Migrate Technology C330 geolocators (GLS) with a wet-dry sensor weighed 3.3 g (0.5% of tropicbird weight) and register if the bird was wet or dry every 6 s. GPS and Axytrek’s were attached to the 6 central tail feathers with Tesa tape while GLS were attached to the tarsus, on the bird’s metal ring with the help of a zip tie.

### Data processing

To test whether adding data from auxiliary sensors improved the accuracy of HMM behavioural inferences, we first processed the wet-dry, accelerometry and TDR data separately before summarizing and matching the information to each GPS position (interpolated to 5-minute intervals). We matched the data forwards (e.g. the value of the wet-dry, accelerometry, and TDR metrics at a GPS position at time *t* summarized the values of the period between *t* and *t* + 1) to be consistent with the calculation of the step and turning angle by the prepData function of the ‘momentuHMM’ package [36]. From wet-dry loggers, we extracted the proportion of time wet between each GPS position. From the accelerometry data, we extracted the proportion of time resting on water, diving, and flapping between each GPS position. From the TDR data, we extracted the number of dives between each GPS position. Further details on device processing methods and their results are in supplementary material S2, S3, and S4.

### Creation of informed dataset

To create an informed dataset of inferred bird behaviour to both semi-supervise and validate the HMM, we combined the information from the wet-dry, accelerometer, and TDR data based on the following conditions to assign positions as foraging, resting, or travelling. These positions are referred to as having a known state.

1. Foraging: diving was identified one or more times in the accelerometer or TDR data stream.
2. Resting: the wet-dry sensor recorded a period as 100% wet, or the accelerometers recorded a period as over 50% on water. No dives were detected in either the accelerometer or TDR data stream.
3. Travelling: the wet-dry sensor recorded a period as 0% wet or the accelerometers recorded a period as 100% flapping. No dives were detected in either the accelerometer or TDR data stream.

### HMMs

We ran two series of HMMs to determine if an increasing percentage of supervision can improve the accuracy of behavioural classifications. The first used only GPS tracks with auxiliary data (151 foraging trips) to determine whether accuracy at high porportions of supervision saturates, while the second used the complete GPS dataset (1084 foraging trips) within which only a small percentage (13.9%) of trips contained auxiliary data to test whether even small auxiliary datasets can improve model accuracy.

All HMMs were implemented in the R package ‘momentuHMM’ [36]. Although GPS loggers were programmed to record positions every 5 minutes, poor satellite reception resulted in gaps in the data (of 6–20 minutes between 1.5% of positions, and over 20 minutes between 0.4% of positions). Therefore, to satisfy model assumptions, GPS data were linearly interpolated to a regularised five-minute sampling frequency to have an equal time period between each position when the gaps were less than 20 minutes long. When gaps were over 20 minutes long, the periods before and after the gaps were handled discretely by the HMMs. HMMs function by identifying underlying latent processes based on the variation in the observed data while also calculating the probabilities of switching from one state to another. When inferring behaviour from animal movement, these models use observed step length and turning angle to infer the underlying (or hidden) behavioural states that drive them [38]. The models separate the modes in a purely data-driven way, by defining the states that best capture the variability in the data. This leaves it to the observer to define a posteriori which state can be used as a proxy for each behaviour based on the estimated movement characteristics (e.g., mean step length and turning angle) of each state. We chose a three-state HMM as a trade-off between model accuracy, interpretability of states, and biological knowledge of the species [39]. States were delineated by the HMM using step lengths and turning angles between positions, and then classified as resting (short step lengths and low turning angles), foraging (mid step lengths and high turning angles), and travelling (longs step lengths and low turning angles). To select appropriate starting values for the models, a k-means clustering algorithm (with k = 3 for the number of states) was used for the state-dependent probability distribution parameters of each data stream [23]. We used a gamma distribution to describe step lengths, and a von Mises distribution with a mean of zero for turning angles. To reduce the risk of models converging at a local rather than global maxima for the maximum likelihood, we reran each model 10 times using a randomization starting values, before selecting the model with the highest maximum likelihood and lowest Akaike Information Criterion (AIC) [36].

### Model validation

To measure how the use of informed data increases model accuracy, we used an iterative approach similar to a k-folds analysis, in which we left out 10 random samples of 10% of the known states to be used as testing datasets, while the remaining 90% of known states were used as training datasets. For the first series of HMMs using only the GPS tracks with auxiliary data, we created models with randomly selected subsets of the known states representing 0 to 75% of this dataset (75% representing the maximum number of known states available for our dataset after setting aside 10% as the testing dataset). For each increase of 5% percent of known states from 0 to 75%, we ran 10 models, using the 10 different random samples of test and training datasets to validate the models. For the second series of HMMs using the complete GPS dataset, we only tested the increase in accuracy between 0 and a maximum percentage of known states (9%) due to computational restrictions and therefore ran 10 models at each of these percentages using the 10 different random samples of test and training datasets.We then decoded the states of each model using the Viterbi algorithm.

**Fig. 1:**
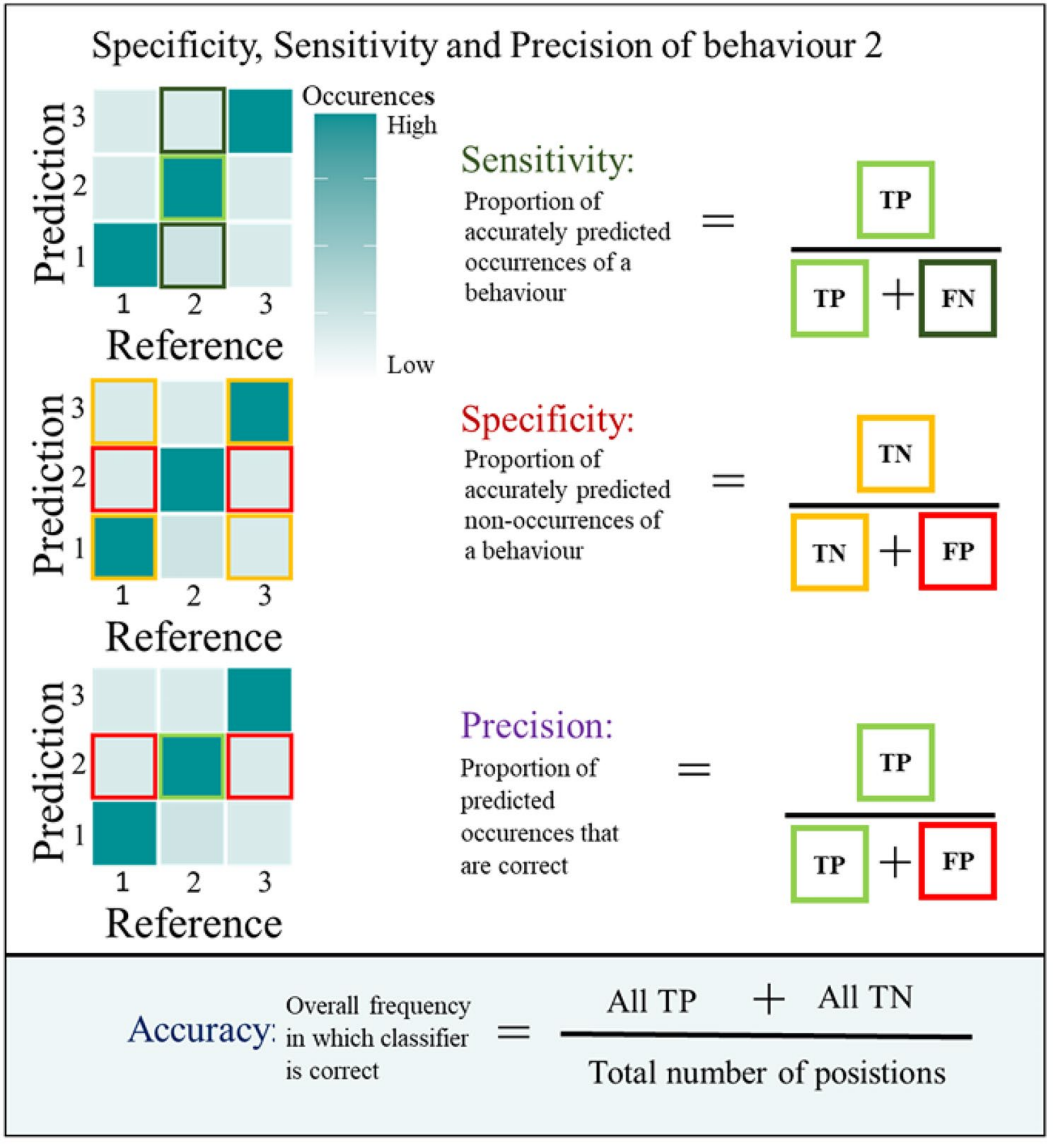
**Example calucaltion of global accuracy, state-wise sensitivity, specificity, and precision using confusion matrices** Example calculation of state-wise sensitivity, specificity and precision for behaviour 2 (in our case foraging) alongside global accuracy using confusion matrices. The confusion matrix colour fills indicate the idealized distribution of the data, with dark squares in the diagonal representing high concentrations of data correctly predicted and clear squares at the edge indicating low concentrations of incorrectly predicted data. TP (true positive – light green outline): number of predictions where the classifier correctly predicts the positive class as positive, TN (true negative – yellow outline): number of predictions where the classifier correctly predicts the negative class as negative, FP (false positive – red outline): number of predictions where the classifier incorrectly predicts the negative class as positive, FN (false negative – dark green outline): number of predictions where the classifier incorrectly predicts the positive class as negative

For each model, we then generated the assigned state confusion matrix to assess overall assignment accuracy using the confusion Matrix function in the ‘caret’ R package [40]. In addition to the overall accuracy we also extracted the class-wise sensitivity, specificity, and precision from the confusion matrices (Fig. 1). These metrics are complimentary and the importance of each will depend on the research questions at hand. Using foraging behaviour as an example, high *sensitivity* of foraging would indicate that most known foraging positions are correctly classified as foraging by the model. However, this does not exclude the possibility of many resting and travelling positions being also misclassified as foraging. To measure this, one uses *specificity*, or the proportion of resting and travelling positions correctly classified as non-foraging. If there is an uneven number of known resting, foraging or travelling positions, even a small proportion of one behaviour misclassified as another can dilute the proportion of correct classifications. Here is when *precision* is needed to determine the proportion of positions classified as foraging that are actually foraging, and not resulting from a misclassification of resting or travelling positions. To compensate for a lack of standardized practices in evaluating and reporting the performance of behaviour classification models [18], we also calculated additional measures of model performance to make it possible to compare our results to as many previous studies as possible (S5).

Finally, to explore if the exclusion of positions with low state classification probabilities improved overall HMM behavioural classification, we used the stateProbs function from the ‘momentuHMM’ package [36] to extract the state classification probability of each position. We then removed all positions with a probability of classification of less than 90%, and evaluated whether this resulted in an increase in the model’s global accuracy and class-wise sensitivity, specificity, and precision.

## Results

We recovered a total of 151 red-billed tropicbird foraging trips with both GPS and auxiliary data, and another 933 trips with GPS data only (Table 1). Within the dataset informed by auxiliary sensors, we were able to classify 83.7% of the GPS positions to either resting, foraging or travelling, representing 10.4% of the complete dataset (including birds equipped only with GPS loggers). After leaving out 10% of positions with known behaviours for model validation, the maximum percentage of supervision within the informed and complete GPS datasets were 75% and 9%, respectively.

**Table 1:** Sensor sample sizes and inferred behavioural states

Auxiliary Sensor(s)	Birds	Deployments	Trips	GPS positions	Known resting	Known foraging	Known travel
**None**	345	420	933	447,346	0	0	0
**ACC + TDR**	20	20	44	23,555	15,642 (66%)	826 (4%)	3743 (16%)
**WD**	26	31	91	31,389	18,767 (60%)	0 (0%)	5973 (19%)
**ACC + TDR + WD**	6	7	16	8539	5850 (69%)	390 (5%)	1962 (23%)
**Total**	**397**	**478**	**1084**	**510,829**	**40,259(8%)**	**1216 (< 1%)**	**11,678 (2%)**

From left to right, auxillary sensor set-up, total number of tracked birds with specified auxillary sensor set-up alongside total sensor set-up deployments, total number of foraging trips, total number of registered GPS positions registered, and the number and percentage of GPS positions with known resting, foraging, and travelling states based on the combination of sensors used. ACC indicates accelerometer, TDR indicates Time Depth Recorder, and WD indicates wet-dry data.

Since tropicbirds were simultaneously tagged with up to 3 auxiliary sensors (across 2 devices), the behaviours of some positions were informed by multiple sensors (Table 2). Using our conservative classification criterion resulted in only 15 positions (out of 8539 positions defined simultaneously by multiple sensors) with incoherent information coming from different sensors (e.g. the accelerometer identified that the bird was resting while the wet-dry sensors identified the bird as flying), therefore the behaviour of these positions was left as unknown for the models. We extracted the highest percentage of GPS positions with known states when animals were tagged with all 3 sensors (wet-dry, accelerometry, and TDR). Accelerometers detected more foraging positions than TDR, recording dives that were shallow (0.78 ± 0.36 m) and short (1.41 ± 0.55 s) (Tables 1 and 2). Wet-dry loggers detected the most resting and travelling positions (Tables 1 and 2). Given the conditions for known states used, we did not predict foraging based on wet-dry data alone nor did we predict resting or travelling based on the TDR data alone (Table 2).

**Table 2:** Total number, percentage, and number of unique positions with behaviours inferred by each auxiliary sensor

Auxiliary Data	Known resting	Known foraging	Known travel
**ACC**	21,490 (67%), unique 16,264	1132 (4%), unique 107	5268 (16%), unique 3761
**WD**	23,995 (60%), unique 18,769	-	7917 (20%), unique 6410
**TDR**	-	1109 (3%), unique 0	-
**Total**	**40,259**	**1216**	**11,678**

The total number, percentage, and the number of positions uniquely identified as known resting, foraging, and travelling based on accelerometry (ACC), wet-dry state (WD) and time depth recorders (TDR). Percentages were calculated based on the total number of GPS positions with each sensor type. The unique number of positions indicates the number of positions that were uniquely identified as a given behaviour by each sensor type given that some positions were informed by more than one sensor simultaneously.

**Fig. 2:**
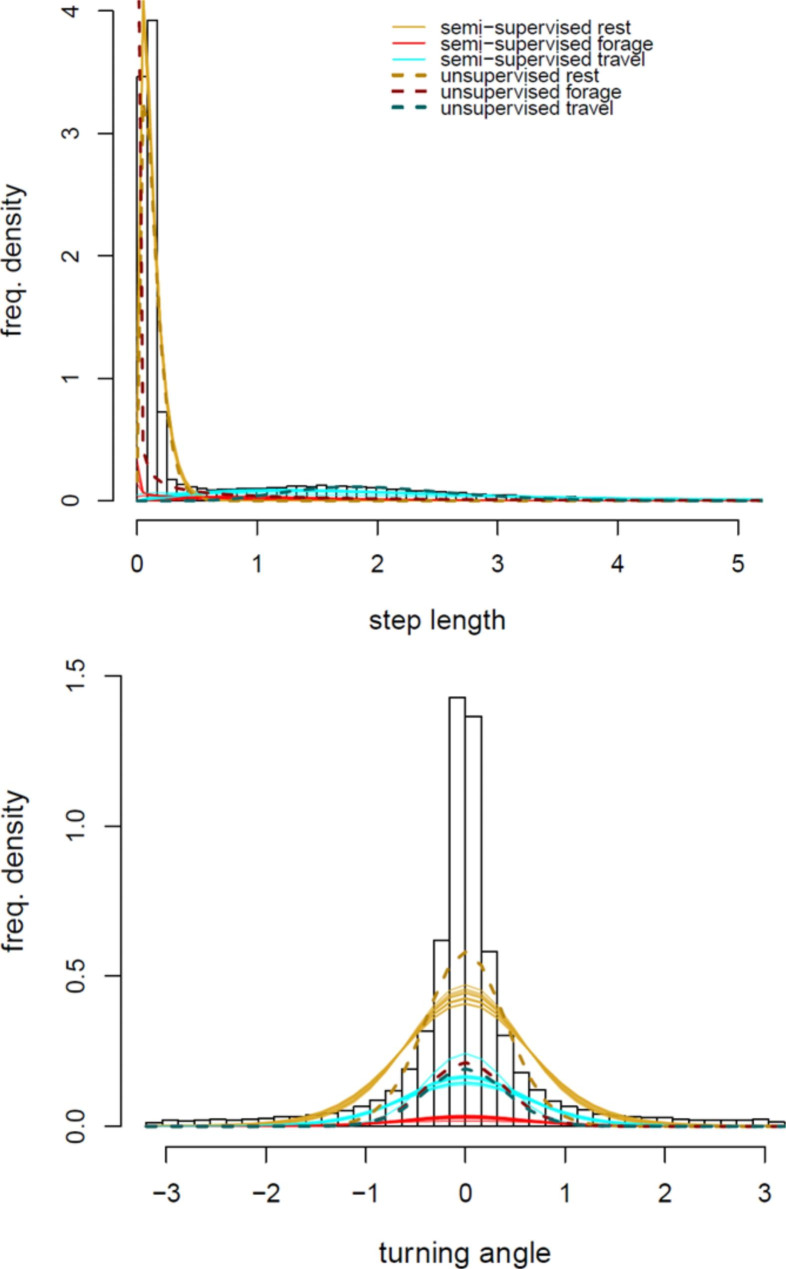
**State-wise distributions of step and turning angle from HMMs without supervision and with maximum supervision (75%)** State-wise distribution of the step length (top) and turning angle (bottom) of resting (yellow), foraging (red) and travelling (cyan). Dashed lines indicate the model with no supervision while solid lines represent each of the 10 iterations of the model with maximum supervision (75%)

As in the auxiliary datasets, the HMM results of all models consistently suggest that tropicbirds spend most of their time resting on water, followed by travelling and foraging (Fig. 2, S4). The transition probabilities between behavioural states also indicate that the probability of remaining in resting from one position to another (0.82 ± 0.05) is much higher than remaining as travelling (0.76 ± 0.05) and foraging (0.59 ± 0.09), and this relationship becomes even stronger with the inclusion of known states (leading to 0.90 ± 0.02, 0.79 ± 0.05, 0.47 ± 0.16 for resting, travelling and foraging respectively with the inclusion of 75% known states, S6). While the turning angle distribution remains similar for the three states with increasing semi-supervision, the distribution of step lengths for the foraging state changed, becoming more overlapped with that of travelling (Fig. 2). This suggests that step length may not be an appropriate metric for separating the behaviour of travelling and foraging.

**Fig. 3:**
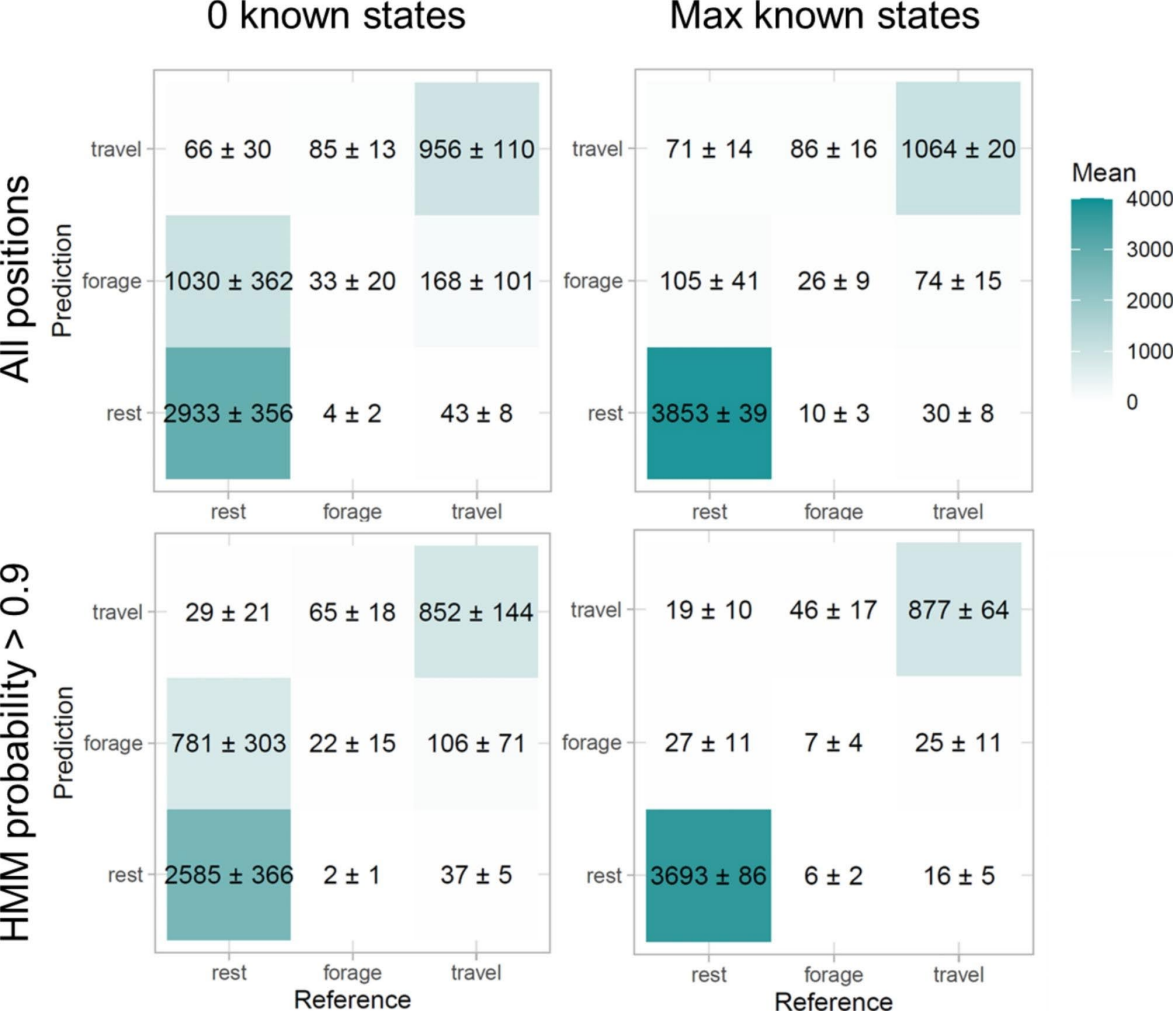
**Confusion matrices of auxiliary data only models** Confusion matrices showing the mean and standard deviation in the number of reference behaviours against model predictions for iterations of the auxiliary data only models with no supervision (left) and with the highest amount of supervision (75%, right). Top row shows all positions while the bottom row shows positions with a classification probability over 0.9

**Fig. 4:**
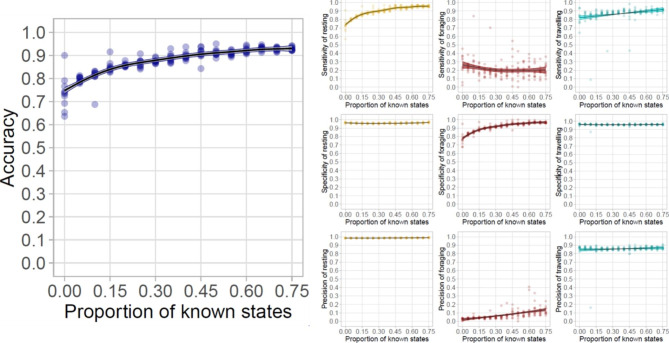
**Accuracy, specificity, sensitivity and precision with incearing known states in the auxiliary data only models** Increase in global accuracy (blue, first column on left), as well as state-wise specificity, sensitivity and precision of resting (yellow; second column), foraging (dark red; third column) and travelling (cyan; forth column, on right) with an increasing proportion of known states included in the auxiliary data only model

**Fig. 5:**
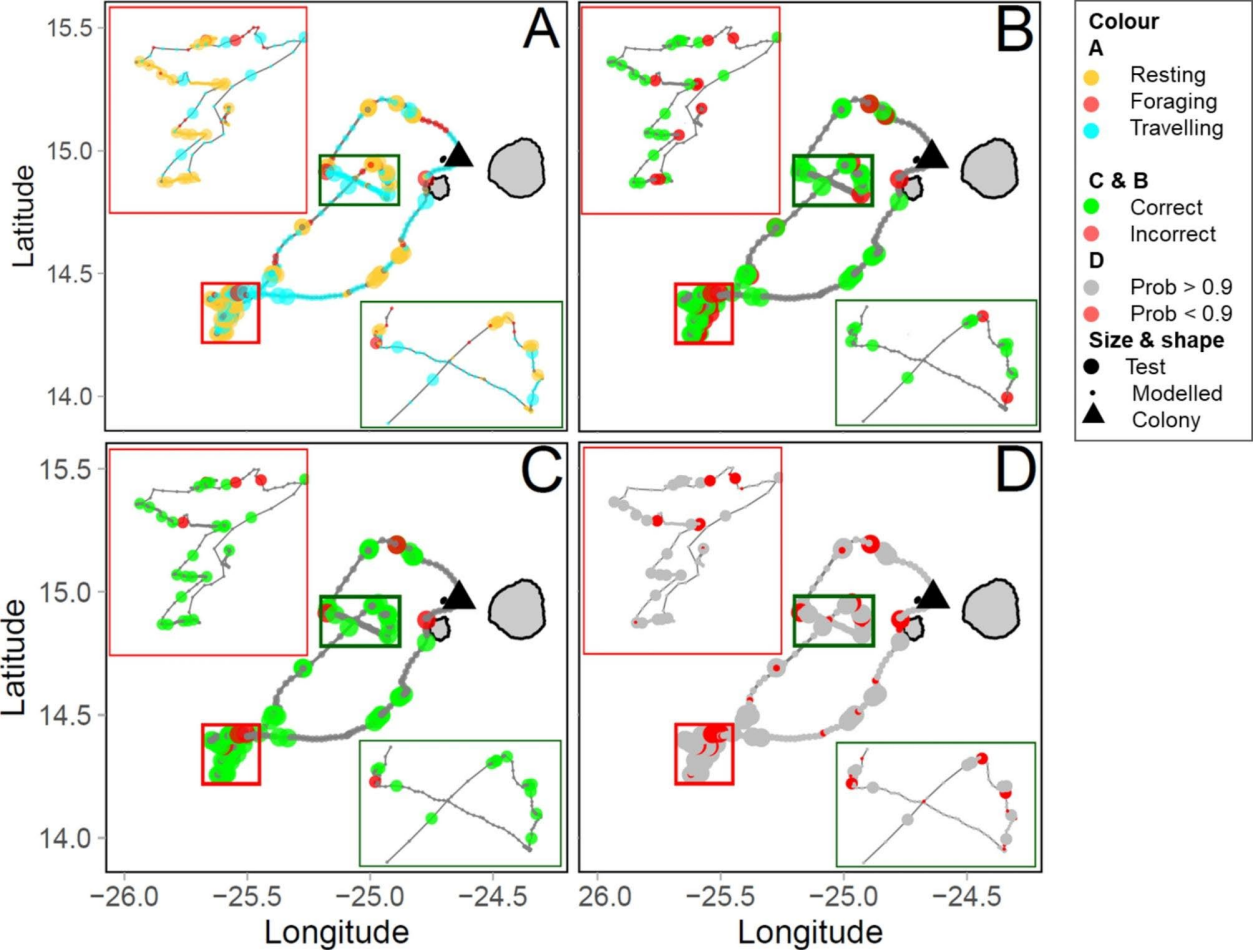
**Example of a foraging trip from the dataset informed with auxiliary data** Large circles indicate positions used as test states to measure accuracy, while small circles represent those included in the model as known states. (**A**) Positions colored by known states (yellow = resting, red = foraging, cyan = travelling). (**B**) Positions coloured by correct (green) or incorrect (red) classification by a HMM with no known states (0%) informed by auxiliary data. (**C**) Positions coloured by correct (green) or incorrect (red) classification by a HMM in which we included 75% known states informed by auxiliary data. (**D**) Positions coloured by the HMM probability of classification, red points have a probability < 0.9 while grey points have a probability > 0.9

In the first series of models using only the data with auxiliary sensors, overall accuracy increased from 0.74 ± 0.07 to 0.93 ± 0.01 when the proportion of included known states increased from 0 to 0.75 (Figs. 3, 4 and 5). This increase in model accuracy was mainly driven by the increase of sensitivity of resting (the proportion of resting correctly identified as such; from 0.73 ± 0.03 to 0.96 ± 0.01) and specificity of foraging (the proportion of non-foraging positions identified as such; from 0.77 ± 0.08 to 0.97 ± 0.01) with a small increase in the sensitivity of travel (from 0.82 ± 0.09 to 0.91 ± 0.01). The specificity of rest and travel of foraging remained relatively stable (going from 0.96 ± 0.01 to 0.97 ± 0.01, and remaining at 0.96 ± 0.01), while the sensitivity of foraging decreased (from 0.26 ± 0.14 to 0.21 ± 0.08). However, these values of sensitivity and specificity were influenced by an uneven number of known resting, foraging and travelling positions, with far more resting and travelling positions than foraging. Therefore, despite the overall improvements to the model, the precision of foraging (the proportion of correctly identified foraging positions) remained low (increasing from 0.03 ± 0.01 to 0.13 ± 0.05), with a high number of resting or travelling positions misclassified as foraging (86 ± 16 and 10 ± 3 respectively) in comparison to the number of positions correctly classified (26 ± 9).

**Fig. 6:**
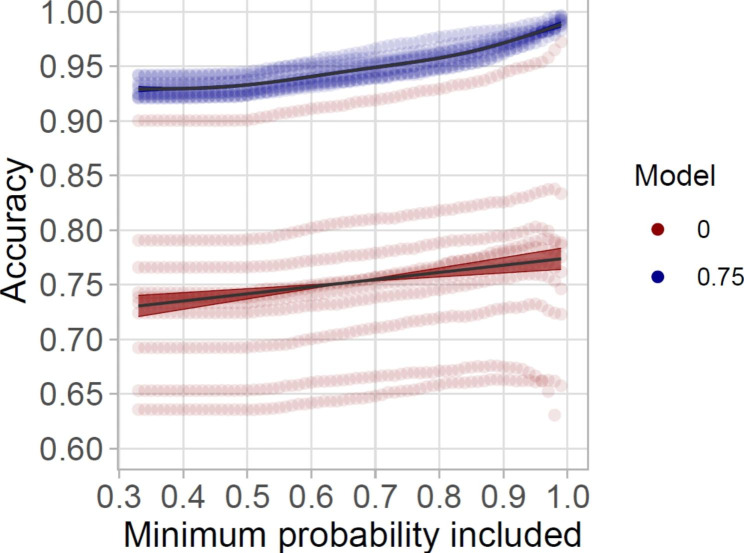
**Increase in model accuracy with the removal of positions with low HMM probability** Increase in model accuracy upon the removal of positions with increasing minimum HMM probability values for behavioural classification. Blue: models with the highest percentage of supervision (75%), red: models without supervision (0%)

Restricting the dataset with HMM classification probability resulted in an increase in model accuracy (Fig. 6), although at the cost of reduced GPS positions for specific behavioural classifications (S7). Foraging positions had the lowest state-wise HMM probability values followed by travelling, and finally resting, resulting in an uneven loss of positions (S7). Moreover, even when reducing the probability of classification to only positions above 0.9, the overall precision of foraging still remained low (0 known states: 0.02 ± 0.02, 0.75 known states: 0.12 ± 0.07) (Fig. 3), suggesting that the number of correctly identified foraging positions was low in comparison to the misclassified resting and travelling positions.

**Fig. 7:**
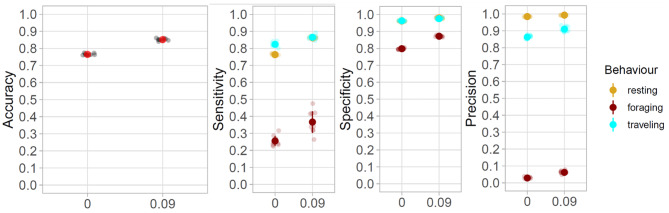
**Increase in global accuracy and state-wise sensitivity, specificity and precision in complete dataset** From left to right, increase in global accuracy (**A**), and state-wise sensitivity (**B**), specificity (**C**), and precision (**D**), of resting (yellow), foraging (dark red) and travelling (cyan) states without and with the maximum proportion of known states in the complete GPS dataset

In the second series of HMMs built using the complete GPS dataset, overall model accuracy increased from 0.77 ± 0.01 to 0.85 ± 0.01 when the inclusion of known states increased from 0 to 9% (Fig. 7, S8). This increase in accuracy was mainly driven by the increase of sensitivity of resting and foraging (from 0.76 ± 0.01 to 0.86 ± 0.01, and from 0.26 ± 0.03 to 0.37 ± 0.06, respectively) and specificity of foraging and travel (from 0.80 ± 0.00 to 0.87 ± 0.01, and from 0.82 ± 0.01 to 0.87 ± 0.01). The specificity of travel and of resting remained relatively stable (from 0.96 ± 0.00 to 0.98 ± 0.00, and from 0.96 ± 0.01 to 0.98 ± 0.00). As in the auxiliary data only model, the precision of foraging, increased with the inclusion of known states but remained low (from 0.03 ± 0.01 to 0.06 ± 0.01), and in comparison to the number of positions correctly classified (44 ± 8), many resting or travelling positions were left misclassified as foraging (5 ± 1 and 71 ± 15, respectively; S8).

## Discussion

We show that semi-supervising HMMs with data from auxiliary sensors, such as accelerometer, TDR, and wet-dry sensors can dramatically improve a state-space model’s global accuracy and state-wise sensitivity and specificity in the classification of GPS tracking data into behavioural states, signifying that the proportion of both true positive and true negative behavioural classification increased. We found that even at small proportions, semi-supervision improved behavioural annotation, although high accuracy (> 0.90) was only reliably achieved with over 32% of known states. Despite this overall increase in accuracy, the foraging behaviours were poorly identified, with state classifications having low sensitivity (0.24 ± 0.17) and precision (0.13 ± 0.05), even with the highest percentage of supervision (75%), indicating a high misclassification rate such that many positions classified as foraging were actually resting or travelling. This suggests that tropicbirds may not use ARS while foraging, but rather forage opportunistically throughout their trips. The exclusion of positions with low HMM probability (< 0.90) alone was not sufficient to improve the classification of the foraging behaviours, further underlining the difficulties in the classification of this behaviour without auxiliary data in species where decision-making is on the go.

### Overall model accuracy

With semi-supervision, the models reached overall accuracy levels similar to previous studies on species with commuting trips (e.g. [17, 41], S1, S5). The overall accuracy was especially high with both semi-supervision and the exclusion of positions with HMM state classification probabilities of < 0.90 (reaching 0.98 ± 0.01), suggesting that combined use of semi-supervision with auxiliary data and thresholds on HMM state classification probability can significantly improve behavioural classification. However, high global accuracy was biased by the correct classification of resting behaviour, which was overly-represented in both the supervised and validation datasets, underlining the importance of state-wise performance measures.

### Behavioural classification and inference

Although semi-supervision improved the overall accuracy of the models, the improvement in the inference was not equal between the three basic behavioural states. While there were strong increases in the sensitivity of resting and the specificity of foraging, the inference of travelling only improved slightly. There was a much steeper decrease of resting positions misclassified as foraging (from 1030 ± 362 to 105 ± 41 with 75% supervision) compared to travelling positions misclassified as foraging (from 168 ± 101 to 74 ± 15 with 75% supervision). This suggests that model semi-supervision mainly helped distinguish between resting and foraging, while confusion between foraging and travelling remained. This is also apparent in the changes of the state-wise distributions of step length with the increase of semi-supervision, with a separation in the distributions of resting and foraging while the distribution of foraging and travelling continued to highly overlap. Without the use of other movement metrics, these overlapping or ‘noisy’ labels essentially cannot be distinguished with HMMs [42]. This suggests that step length is not a good movement metric for separating foraging and travelling behaviour in this species, and highlights the challenges associated with delineating opportunistic feeding events in seabirds foraging on the wing.

Despite improvements to overall accuracy, we found much lower sensitivity and precision of foraging than what was previously reported from studies using HMMs to classify the foraging behaviour of other seabirds [17, 23, 41]. The sensitivity of foraging for the semi-supervised models was low and was not improved by semi-supervision, declining from from 0.26 ± 0.14 to 0.21 ± 0.08 with the highest percentage of supervision (75%), suggesting that many foraging positions were undetected and that this number is not reduced by semi-supervision. Moreover, the precision of foraging behaviour increased from 0.03 ± 0.01 to 0.13 ± 0.05 with the highest percentage of supervision (75%), but did not saturate, indicating that this level of semi-supervision was insufficient to prevent erroneous inference of foraging states.

Difficulty in correctly classifying foraging positions may can be discussed at both model and ecological levels. At the model level, this was caused by a large overlap between the state-wise distribution of foraging and that of the other behaviours, signifying that, based on step length and turning angle alone, HMMs were unsuccessful at distinguishing the signal of foraging from the other behaviours [42]. At the ecological level, this overlap between behavioural signals may stem from the distribution of tropicbird’s prey and foraging strategy compared to other non-tropical seabirds, such as large shearwaters, auks or gannets [17, 23, 41]. Tropicbirds are offshore specialists that mainly forage on flying fish [28], in waters of low-productivity [43, 44], making their distribution highly unpredictable both in time and space. Such patterns are possibly driven by the low predictability of prey distributions in tropical oceans, resulting in low foraging site fidelity and a prominence of looping trips, as observed in many other tropical species [24, 25, 45–47]. This contrasts with the commuting trips of non-tropical seabirds who concentrate foraging in predictable areas associated with high productivity [48]. Some tropical species often forage opportunistically, with prey-capture attempts occurring within directional transit [24, 49], making it difficult for behavioural models to differentiate foraging from travelling locations. Although opportunistic foraging appears to cause a higher classification error for foraging compared to other behaviours in tropical sullids [16, 26, 37], the error rate in tropicbirds is particularly high, suggesting that this species may use opportunistic foraging more frequently than other tropical species.

If not addressed, the low sensitivity and precision of foraging in these models can have important implications in conservation and management decisions. Foraging areas are often the target of spatial management plans because of their ecological importance for species, and therefore their correct identification is critical [1, 9, 10]. In models with low foraging sensitivity, many foraging positions are going undetected, suggesting that in theory these models may underestimate total foraging ranges. However, previous studies with high misclasssification rates have demonstrated strong spatial overlap between true foraging positions extracted from TDRs and modelled foraging areas [37, 41], suggesting this may not be an issue in practical terms. This may be because opportunistic foraging positions are well dispersed throughout trips, resulting in a higher than usual overlap between foraging and home range areas [25]. More importantly, in this study the precision of foraging also remained low, leaving a high percentage of resting and travelling positions erroneously identified as foraging. This may have important implications for habitat modelling studies, since resting and travelling positions misclassified as foraging may be obscuring important behaviour-specific habitat relationships [50] and potentially time-activity budgets [51].

### Improving behavioural classification for opportunistic foragers

Whilst semi-supervised learning can improve association between observed movement metrics and desired behavioural states, limitations exist. In such instances, the inclusion of additional auxiliary sensors, such as TDR, accelerometers, and/or cameras, may be necessary across the full dataset to identify less frequent behaviours such as prey-capture attempts, and achieve satifcatory model performance. If the sampling resolution of the GPS positions is greater than the duration of certain behaviours, the signal of these behaviours may be obscured by others associated to the same GPS fix, and thus the application of auxiliary sensors may need to be coupled to increases in the temporal resolution of GPS locations. Although HMMs have been shown to be relatively robust against reductions in resolution in comparison to other methods, such as deep learning [16, 17], the infrequency of diving behaviour may make it especially difficult for the models to correctly identify [52]. In our study, dives only lasted 1.4 ± 0.6 s seconds and were infrequent and dispersed (just 1.2 ± 1.3 dives per GPS position, and only 22% of dives were recorded within the same or in adjacent GPS positions), suggesting that foraging may be obscured by resting and travelling if dive-specific auxiliary data is not available. Similar observations have been made in the attempt to distinguish mating behaviour in GPS-tracked deer [53] or in the differentiation of natural and non-natural foraging in seabirds [3]. In these cases, the addition of more complexe auxiliary sensors (such as cameras, TDRs, and accelerometers etc.) may be needed to truly identify these particular behaviours. Auxiliary devices have been used in combination with GPS data to identify foraging behaviours in many seabirds and seals, which may otherwise be impossible [3, 32, 54, 55].

In the case of opportunistic foragers, such as red-billed tropicbirds, the identification of foraging habitat based solely on dives may underestimate the foraging area used by these species. If prey-capture attempts occur opportunistically within directional transit, it may be ineffective to separate directional movements from foraging. This is reflected by the proportionally small improvement of model classification when it came to separating foraging from travelling with semi-supervision. The relative homogeneity of tropical oceans may render the identification of foraging behaviour meaningless, since birds actually seem to search for prey over the entire looping trips. In this regard, teasing apart resting from non-resting behaviour may be enough for subsequent analyses of foraging habitat use and preferences in opportunistic foragers.

### Guidance for the implementation of semi-supervised behavioural classification

Foremost, semi-supervised learning can improve associations of observed movement metrics with desired behavioural states, but, only if the chosen metrics are distinct for each of the states. If the metrics highly overlap (as the step lengths of foraging and travelling did in our study), overall improvements will be limited. Therefore, it is important to choose the right sensors, recording frequency, and movement metrics to answer specific research questions a priori to undertaking the research in question. This, of course, is easier said than done, since the choice of such metrics will also depend on the ecology and behaviour of the species in question, which may be unknown to the researcher before the commencement of the study. Therefore, we suggest combining both semi-supervision and model validation when possible, to make sure that the assumptions of the ecology of the species made at the beginning of the study are correct, and that movement metrics are accurately identifying the chosen behaviours.

Although all auxiliary sensors helped improve model accuracy, each sensor came with its own advantages and disadvantages, which vary with the specific study question and ecology of study species. Here, wet-dry loggers generated the largest number of positions with known behaviours alone, primarily because tropicbirds spend the majority of time resting on water [28]. In seabird species that spend more time on the wing, wet-dry sensors may detect fewer resting positions, but can still be used to identify potential prey capture attempts within foraging [3, 23]. TDR loggers, on the other hand, gave accurate measures of foraging attempts but could not detect when the bird was resting or travelling, and recorded fewer overall dives than accelerometers, possibly because of missed shallow dives [56] or the capture of flying fish in air [30]. In species with deeper and more complex dives, TDR devices can greatly improve behavioural classifications [57].

Accelerometers where the only auxiliary sensor that allowed for the detection of all three behavioural states. However, the complexity of processing accelerometer data is much higher than wet-dry loggers and TDRs. Transforming accelerometer data into behavioural states required the additional step of extracting periods of flapping, diving, and resting from the accelerometer signals, a process which in our case, was semi-supervised by both WD and TDR data. This added an additional layer of complexity and potential error to modelling the raw accelerometry data while also highlighting the importance of WD and TDR devices in identifying behaviour. Therefore, the selection of auxiliary sensors to use for a given study should consider both the complexity of the study question, and the ecology of the study species.

### Future research

In the present study, we highlight the benefits of semi-supervision in HMMs while creating awareness of possible misclassifications and the importance of cross validation. Whilst using real world tracking data allowed us to demonstrate the applied ramifications of this in a biological context, we were unable to measure the absolute increase in accuracy related to semi-supervision and suggest that a follow-up simulation study could greatly improve our overall understanding of limitations of HMMs. Such a study would comprise of creating datasets with increasing levels of overlap between state distributions, and measuring how HMMs of these datasets react to increasing semi-supervision. This would allow researchers to create guidelines based on the initial distribution of data to understand if, and/or how much semi-supervision is needed to improve the overall classification. Since data would be simulated, issues relating to uneven datasets and possible introduced errors from inferring the known behaviours from auxiliary datasets would be eliminated. Such an analysis could also be used to make inferences on the limitations of HMMs in situations beyond movement ecology, and we recommend this as a more generalised future study.

## Conclusions

Semi-supervision increased model accuracy, even when positions with inferred behaviours represented a small proportion of the dataset. This increase was uneven among the three basic behaviouralstates, with stronger increases in the sensitivity of resting and the specificity and precision of foraging, while travelling remained relatively stable. Despite these improvements, the behavioural inference levels of foraging remained low compared to those of species using commuting foraging trips, and may not be enough for the analysis of foraging habitat use and preferences. Precaution should be taken in the identification and use of foraging behaviour states in opportunistic foragers, such as species searching for prey across a homogeneous environment. The nature of the foraging behaviour of species foraging on the go may lead to an over-fitted identification of foraging behaviour. Indeed, we suggest that in this type of species, distinguishing resting from non-resting behaviours should be enough for subsequent analyses of foraging habitat use and preferences. However, even in these cases, the use of semi-supervision can greatly improve behavioural inferences and the choice of auxiliary sensor(s) will depend on the specific ecology of species, deployment logistics, processing time, and costs.

## Electronic supplementary material

Below is the link to the electronic supplementary material.

Supplementary Material 1

Supplementary Material 2

Supplementary Material 3

Supplementary Material 4

Supplementary Material 5

Supplementary Material 6

Supplementary Material 7

Supplementary Material 8

## Data Availability

GPS Tracking data is available in the Seabird Tracking Database of Birdlife International. All the R workflow is available at https://github.com/SarahSaldanha/Semi_supervised_HMM.

## List of Abbreviations

HMM: Hidden Markov Models
TDR: Time Depth Recorders
GPS: Global Positioning System
ARS: Area Restricted Search
OFT: Optimal Foraging Theory
EmbC: Expectation-maximization Binary Clustering
RST: Residence in Space and Time
FPT: First Passage Time
TP: True Positive
TN: True Negative
FP: False Positive
FN: False Negative
ACC: Accelerometry
WD: Wet-dry
