# Supplementary material for: Animal behaviour on the move: the use of auxiliary information and semi-supervision to improve behavioural inferences from Hidden Markov Models applied to GPS tracking datasets
Source: Mov Ecol. 2023 Jul 24;11:41. doi: 10.1186/s40462-023-00401-5 (PMC10367325; doi:10.1186/s40462-023-00401-5)
Supplement: Supplementary file 2 — Supplementary Material 2 [file 40462_2023_401_MOESM2_ESM.docx]

**S2**

**Data Processing**

*GPS*

We processed and standardised GPS tracks from 397 breeding red-billed tropicbirds. The GPS tracks were cleaned of erroneous positions using a speed filter (30m/s) and split into discrete foraging trips with periods in the nest between trips omitted. Incomplete trips were also included in the analysis if they were over 30 minutes long. Although GPS tracks were set to record positions every 5 minutes, poor satellite reception resulted in gaps in the data. Therefore, we used linear interpolation to regularise the data to a common time interval of 5 minutes for every segment of data with gaps less than 20 minutes before fitting the behavioural models.

*Accelerometers*

We recovered 27 axy-trek devices from 26 individuals and split these into 60 foraging trips based on the GPS positions (Table 1). These devices contained both a tri-axial accelerometer which recorded acceleration in the surge (X, forwards/backwards), heave (Z, dorso/ventral) and sway (Y, side to side) axis at a frequency of 25 Hz and a TDR sensor which recorded pressure at a frequency of 1Hz.

We classified the accelerometery signals into behavioural states with a random forest model (RF) using the ‘randomForest’ package in R (Liaw & Wiener, 2002). To build the RF model, we used a training dataset with supervised behavioural classifications to predict the behaviours of the complete accelerometer dataset. To create the training dataset, we used a subset of 16 trips from 6 individuals which had wet-dry data in addition to accelerometery and TDR data (Table 1). Using the wet-dry and TDR data to validate our classifications, we manually classified each signal into 3 behaviours in the software Framework4 (Walker et al., 2015): flying (dry, high acceleration in X and Z), on water (wet, low acceleration in X and Z) and diving (wet, high acceleration in X and Z and high pressure) based on the patterns in static acceleration for X, Y and Z as well as their derived variables pitch and roll, and, finally, the wet-dry and TDR pressure data.

Since the device was deployed on the tail of the bird and not in the back, the pitch and roll, which usually indicate whether the bird was standing vertically or horizontally and whether the bird was positioned with its dorsal side up or down, respectively, more likely indicated strong halting or turning. Pitch and Roll were calculated based on the following equations:

$$Pitch= Arctan\sqrt{\frac{X}{Y^{2}+Z^{2}}}*\frac{180}{\pi}$$

$$Roll= Arctan\sqrt{\frac{Y}{X^{2}+Z^{2}}}*\frac{180}{\pi}$$

To reduce the number of false positives in the random forest model, we only manually annotated dives when there was a visual change in pressure recorded by the TDRs and a change from dry to wet. However, shallow dives are underestimated by TDR (Cianchetti-Benedetti et al., 2017) and wet-dry data was recorded at a relatively coarse intervals (6s), therefore we expected more dives to be predicted by the model than those identified manually.

Next, we segmented both the training dataset and the complete dataset into variable segment lengths using a change point model using the R package ‘cpm’ (Ross & Gordon, 2020) following the methods described in Born et al. 2014. We chose to use variable-time and not fixed-time segments to assign boundaries between behavioural classes since variable-time segments have been shown to improve the classification of some behaviours (Born et al. 2014).

For each of these segments, we then calculated various metrics to be fed into a RF model. We calculated the mean, standard deviation, maximum, minimum, cumulative positive, cumulative negative and cumulative absolute values of the X, Y, Z, pitch, roll and depth signals of the accelerometer data. In addition, we calculated two measures of dynamic body acceleration (DBA) for each of the sections: the overall dynamic body acceleration (ODBA) and vectorial dynamic body acceleration (VeDBA). These were calculated by taking a running mean of the raw data from each accelerometer axis across a 2-sec period to calculate the static acceleration, and then subtracting the static acceleration values from the raw acceleration values for that time period. ODBA and VeDBA were then calculated as follows:

$$VeDBA=\sqrt{{(a}_{X}^{2} {+a}_{Y}^{2}+a_{Z}^{2}}$$

$$ODBA= a_{X}+ a_{Y}+ a_{Z}$$

We then matched the behavioural classifications of the training dataset to each of the segments and ran a RF model to predict the behaviours of the unclassified accelerometer data. We used 1000 trees and optimized the number of predictor variables that were randomly selected at each node using the function ‘mtry’ from the ‘randomForest’ package in R (Liaw & Wiener, 2002). This function ran separate models with 0 to 15 predictor variables/node, allowing us to select the number of predictor variables which resulted in the lowest out of the bag (OOB) error. The OOB error is a built-in test of accuracy, in which bootstrapping is used to calculate classification errors within the RF models and estimates the overall accuracy of the model by holding back and comparing the classification of random subsets of the training dataset, selected with replacement. We also estimated the importance of the predictor variables using the function ‘varImpPlot’ from the ‘randomForest’ package in R (Liaw & Wiener, 2002) which measures the mean decrease in accuracy based on the change in the prediction error when the OOB data for that variable are re-arranged and all other variables are left unaffected. We classified the behaviours of the complete accelerometery dataset using the ‘predict’ function. To reduce the amount of misclassification, we only assigned the behaviours to a segment when the probability of said behaviour was over 0.65. This threshold was selected as a trade-off between accuracy and the proportion of unclassified segments.

We then matched the accelerometery data to each GPS position by summarizing the proportion of time flying, on water and the total number of dives for each time period between interpolated GPS positions.

*TDR*

The time-depth recorder (TDR) data of the 27 Axy-treck tags were also used to identify diving identify dives based on the pressure sensor alone. Since the tags were deployed over a long period, the atmospheric pressure at sea-surface (Pm in mBar) was expected to vary greatly between trips. Therefore, we estimated Pm for each trip as the mode [pressure] and calculated depth (D in m) using the relationship: D = 0.01 × (Pm − Pa), where 1 mBar pressure difference corresponds to 0.01 m depth difference. We corrected for surface drift using the zero-offset correction of the diveMove package in R (Luque & Fried, 2011). The pressure sensors were very sensitive and small differences in depth may result from preening, fast turns, and takeoff instead of dives. Therefore, only dives ≥0.2 m were considered.

We then matched the TDR data to each GPS position by counting the total number of dives for each time period between the interpolated GPS positions.

*Wet-dry*

Twenty-five individuals with GPS tracks were outfitted with Migrate GLS, resulting in 107 foraging trips with at least both types of data. Using conductivity, these devices detect whether they are in water or not every 6 seconds and record the timing of transitions from wet to dry or from dry to wet. We matched the wet-dry data to each GPS position by summarizing the proportion of time wet (PropWet) for the periods of time between each interpolated GPS position. We then eliminated PropWet data when the time gap from one point to the next was over 5 minutes (caused by gaps in the GPS data) (0.5% of the positions).

**References:**

Cianchetti-Benedetti, M., Catoni, C., Kato, A., Massa, B., & Quillfeldt, P. (2017). A new algorithm for the identification of dives reveals the foraging ecology of a shallow-diving seabird using accelerometer data. *Marine Biology*, *164*(4). https://doi.org/10.1007/s00227-017-3106-0

Liaw, A., & Wiener, M. (2002). *Classification and Regression by RandomForest*. https://www.researchgate.net/publication/228451484

Luque, S. P., & Fried, R. (2011). Recursive filtering for zero offset correction of diving depth time series with GNU R package diveMove. *PLoS ONE*, *6*(1). https://doi.org/10.1371/journal.pone.0015850

Ross, J., & Gordon, M. (2020). *Package “cpm.”* www.gordonjross.co.uk,

Walker, J. S., Jones, M. W., Laramee, R. S., Holton, M. D., Shepard, E. L., Williams, H. J., Scantlebury, D. M., Marks, N. J., Magowan, E. A., Maguire, I. E., Bidder, O. R., di Virgilio, A., & Wilson, R. P. (2015). Prying into the intimate secrets of animal lives; software beyond hardware for comprehensive annotation in ‘Daily Diary’ tags. *Movement Ecology*, *3*(1), 29. https://doi.org/10.1186/s40462-015-0056-3
