# Supplementary material for: Animal behaviour on the move: the use of auxiliary information and semi-supervision to improve behavioural inferences from Hidden Markov Models applied to GPS tracking datasets
Source: Mov Ecol. 2023 Jul 24;11:41. doi: 10.1186/s40462-023-00401-5 (PMC10367325; doi:10.1186/s40462-023-00401-5)
Supplement: Supplementary file 4 — Supplementary Material 4 [file 40462_2023_401_MOESM4_ESM.docx]

**S4**

Random Forest Results

We classified 1663, 6076 and 19332 segments within the training dataset as dives, flapping and on water, respectively based on the wet-dry and TDR data. A preliminary inspection of the mean and standard deviation of all 46 of the random forest coefficients suggested differences in the metrics of segments classified with different behaviours (S3).

The OOB error for the random forest created from the training dataset was lowest with 7 variables tried at each split and therefore this model was used to predict the behaviours of the full dataset. The OOB error was 18% with the highest class error associated with diving behaviour (42%) followed by flapping (0.5%) and finally on water (0.06%). The most important predictors of behaviour in this model were the maximum heave, minimum depth, and mean depth of the segments (Figure 1).

After predicting the behaviours of the full dataset and removing classifications with a probability of less than 0.65, 58% of the accelerometery dataset was classified as either dive, flapping or on water (Table 1).

**Table 1. The total and per trip mean and standard deviation of the number of segments and proportion of time spent in each of the behaviours classified by the Random Forest model.**

**
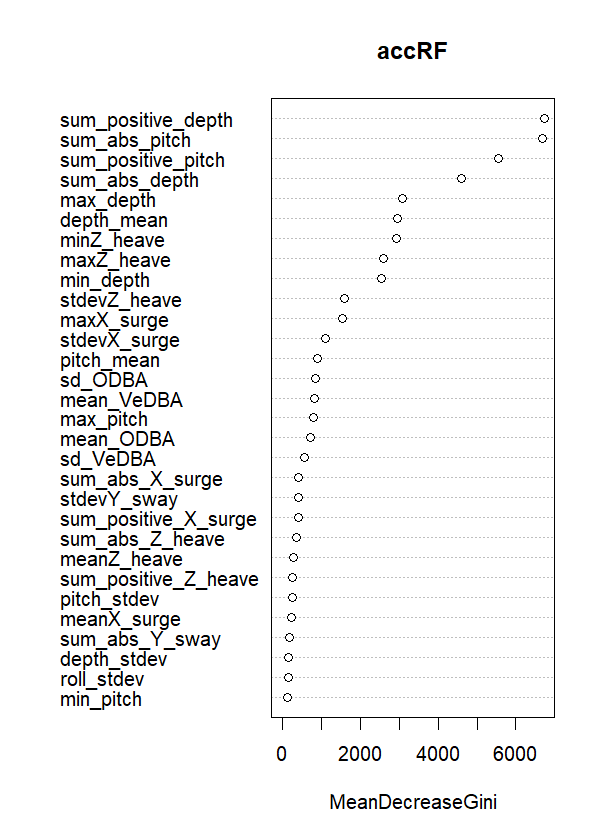
Figure 1. Results of the varImpPlot showing the importance of each variable in the random forest model**
