# Supplementary material for: Animal behaviour on the move: the use of auxiliary information and semi-supervision to improve behavioural inferences from Hidden Markov Models applied to GPS tracking datasets
Source: Mov Ecol. 2023 Jul 24;11:41. doi: 10.1186/s40462-023-00401-5 (PMC10367325; doi:10.1186/s40462-023-00401-5)
Supplement: Supplementary file 6 — Supplementary Material 6 [file 40462_2023_401_MOESM6_ESM.docx]

Table 1. Mean and standard deviation of HMM transition probabilities between resting, foraging and travelling from the 10 iterations of models with 0 known states

|  | rest | forage | travel |
| --- | --- | --- | --- |
| rest | 0.82 ± 0.05 | 0.12 ± 0.03 | 0.05 ± 0.02 |
| forage | 0.23 ± 0.05 | 0.59 ± 0.09 | 0.15 ± 0.05 |
| travel | 0.099 ± 0.05 | 0.14 ± 0.06 | 0.76 ± 0.05 |

Table 2. Mean and standard deviation of HMM transition probabilities between resting, foraging and travelling from the 10 iterations of models with a maximum proportion of known states (0.75)

|  | rest | forage | travel |
| --- | --- | --- | --- |
| rest | 0.90 ± 0.02 | 0.05 ± 0.01 | 0.05 ± 0.01 |
| forage | 0.22 ± 0.17 | 0.47 ± 0.16 | 0.23 ± 0.14 |
| travel | 0.12 ± 0.03 | 0.10 ± 0.04 | 0.79 ± 0.05 |
