# Supplementary material for: Animal behaviour on the move: the use of auxiliary information and semi-supervision to improve behavioural inferences from Hidden Markov Models applied to GPS tracking datasets
Source: Mov Ecol. 2023 Jul 24;11:41. doi: 10.1186/s40462-023-00401-5 (PMC10367325; doi:10.1186/s40462-023-00401-5)
Supplement: Supplementary file 7 — Supplementary Material 7 [file 40462_2023_401_MOESM7_ESM.docx]

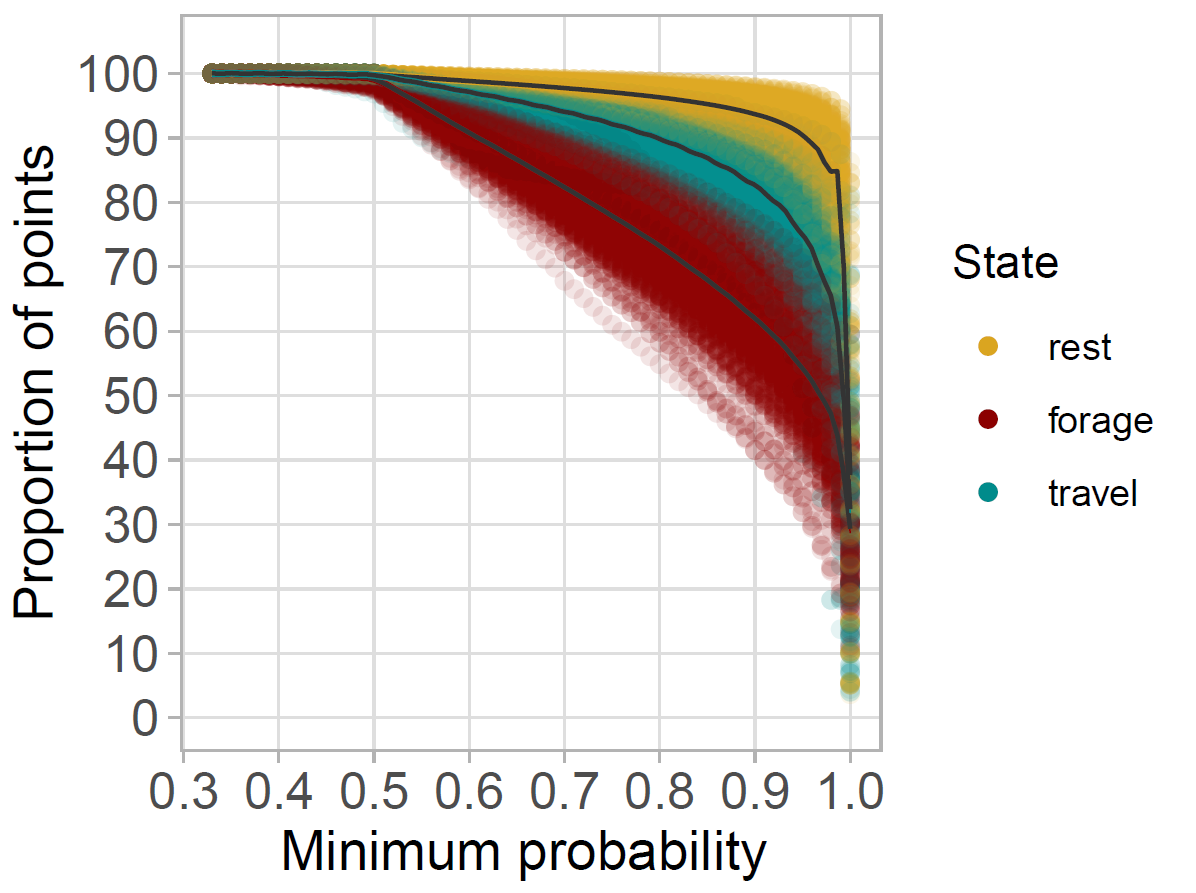


**S7. Sample loss with removal of positions with low HMM probability**

Proportion of GPS positions remaining in the dataset upon removal of positions with increasing minimum HMM probability values for behavioural classification.
