# Supplementary material for: Animal behaviour on the move: the use of auxiliary information and semi-supervision to improve behavioural inferences from Hidden Markov Models applied to GPS tracking datasets
Source: Mov Ecol. 2023 Jul 24;11:41. doi: 10.1186/s40462-023-00401-5 (PMC10367325; doi:10.1186/s40462-023-00401-5)
Supplement: Supplementary file 8 — Supplementary Material 8 [file 40462_2023_401_MOESM8_ESM.docx]

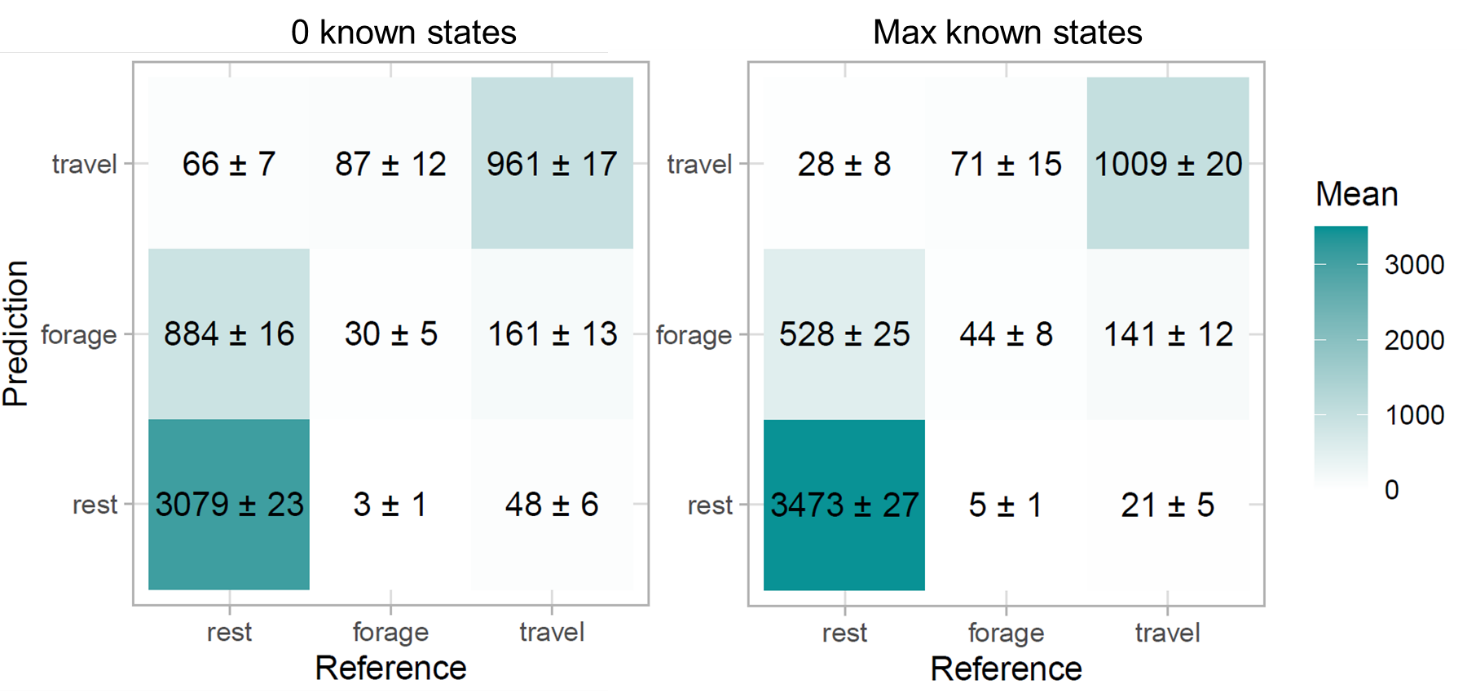


**S8: Confusion matrices of complete GPS HMMs**

Confusion matrices showing the mean and standard deviation of the number of reference behaviours against model predictions for iterations of the complete GPS HMMs with no supervision (left) and with the highest amount of supervision (9%, right).
